# A global survey of national oral health policies and its coverage for young children

**DOI:** 10.3389/froh.2024.1362647

**Published:** 2024-04-05

**Authors:** Balgis Gaffar, Robert J. Schroth, Moréniké Oluwátóyìn Foláyan, Francisco Ramos-Gomez, Jorma I. Virtanen

**Affiliations:** ^1^ Department of Preventive Dental Sciences, College of Dentistry, Imam Abdulrahman bin Faisal University, Dammam, Saudi Arabia; ^2^ Departments of Preventive Dental Science, Dr. Gerald Niznick College of Dentistry and Departments of Pediatrics & Child Health and Community Health Sciences, Max Rady College of Medicine, Rady Faculty of Health Sciences, University of Manitoba, Manitoba, MB, Canada; ^3^ Department of Child Dental Health, Obafemi Awolowo University, Ile-Ife, Nigeria; ^4^ UCLA Center for Children’s Oral Health (UCCOH), University of California, Los Angeles, Los Angeles, CA, United States; ^5^ Faculty of Medicine, University of Bergen, Bergen, Norway; ^6^ Institute of Dentistry, University of Turku, Turku, Finland

**Keywords:** healthy policy, universal health coverage, oral health, dental care, child

## Abstract

**Background:**

There is no accessible information on countries with oral health policies. The purpose of this study was to identify World Dental Federation (FDI) member countries with oral health policies and their scope and extent of coverage of oral health care for young children.

**Methods:**

This international survey recruited chief dental officers, oral health advisors to national ministries of health, and other key informants of the 158 FDI member countries between December 2020 and December 2021. The survey tool was administered online to the study participants. Key questions explored the following outcome measures: countries with oral health policies; the thrusts of the oral health policies; policy thrusts targeting young children; and dental care plans as a component of a universal health care plan. Descriptive statistics were conducted to determine the number of countries with any of the study outcome measures and coverage per country.

**Results:**

Sixty (38%) of the 158 FDI member-countries responded to the survey. Forty-eight (55.2%) of the 60 countries had a national oral health policy document or position statement on oral health; 54 (62.1%) countries had plans on universal health care, and 42 (48.3%) included dental care within their universal health care plan. The most common policy thrusts addressing the oral health needs of children were the promotion of oral hygiene (71.7%), provision of fluoride products for children (53.3%), collaboration with primary care providers (35%), and prenatal oral health education (50%). There were differences in the scope of oral health care coverage and the coverage for young children between continents as well as between countries. Europe had many countries with children-friendly oral health policy coverage.

**Conclusions:**

About half of the surveyed countries had a national oral health policy. There were variations in the scope of oral health care coverage, particularly for young children, both between continents and among individual countries. These findings underscore the importance of understanding the landscape of oral health policies globally. Such insights can help inform targeted interventions to enhance oral health policies, thereby contributing to improved oral health outcomes on a global level.

## Introduction

Although oral health is an integral part of general health, global and national policy-making processes often fail to include oral health and oral health policies, as oral diseases are considered non-life-threatening diseases (1). Not only is oral health an essential component of health and a fundamental human right (2); it contributes to physical, emotional, spiritual, intellectual, and social well-being, and has a significant impact on the quality of life (3).

Macro and micro-level factors ameliorate the occurrence of oral diseases among individuals. One central macro-level factor affecting oral disease is oral health policy, particularly at the national level (4, 5). Health policy constitutes the drive of decisions for improvement of health status of people are made. Comprehensive oral health care policies help to improve individuals' access to services and resources that enhance their oral health (1). Health policies also provide templates for developing healthcare delivery guidelines that promote access to quality health care for patients and support administrators in building efficient healthcare systems (6). When patients are informed about healthcare policies, they learn more about their rights and are empowered to advocate for change and equity when needed (7). Developing an oral health policy is a continually dynamic process, which is human and capital intensive (6).

In its report “Vision 2030—Delivering Optimal Oral Health for All”, the World Dental Federation (FDI) calls for efforts at the global level to achieve empowered, evidence-based, integrated, and complete oral healthcare by 2030 (8). The three priority initiatives include creating a robust oral health workforce, integrating oral and general healthcare, and providing universal coverage for oral health (8). FDI member countries that adopt this vision may have to develop or revise existing oral health policies, guidelines or strategies. There is currently no accessible publication that has mapped existing national oral health policies on a global level. Mapped policies are tools to inform critical thinking and planning, including resource investment, as it can provide an overview of the whole system the policy supports (9).

Childhood oral problems can have a significant impact on the lives of children and their parents, and possibly for the whole life span of affected individuals (10–13). The quality of life for persons affected by oral diseases is significantly reduced because they are among the most common diseases in the world and have substantial health and financial consequences. Chronic untreated dental diseases have serious personal repercussions that can include unrelenting pain, sepsis, lower quality of life, missed school days, disturbance of family life, and decreased productivity at work (14). Families and healthcare systems have a heavy financial burden due to the expense of treating oral diseases (15). Exposure to risk factors including environmental factors at a young age exacerbates these consequences. As such there is a need for public policies that address the factors associated with childhood oral problems and tailor preventive interventions accordingly.

The prevalence and severity of oral conditions and oral health care needs are greater among children from disadvantaged, racial/ethnic, lower socioeconomic groups as well as in those with special needs (16). These disparities in oral health are multifaceted and are linked to availability and affordability of dental services as well as access to preventive care (17).

Early childhood caries (ECC) is the most common oral disease in children worldwide (18). It has long term consequences on the oral and general health as well as quality of life of the child (19). Yet ECC is preventable when effective policies are instituted to promote the prevention and control of oral diseases in infants, toddlers, and preschool children through provision of pre- and post-natal oral healthcare (20, 21).

This study aims to map FDI member countries with oral health policies, provide a snapshot of the content of the oral health policies, and identify the scope and coverage of oral health care for young children.

## Methods

### Study design

This survey was conducted between December 2020 and December 2021.

### Study participants

The target participants were chief dental officers of FDI member countries listed on the FDI website, oral health program directors associated with respective Ministries of Health (MoH) and key informants in FDI member countries. FDI's membership comprises approximately 200 national member dental associations and specialist groups in some 158 countries (https://www.fdiworlddental.org/members). Countries were included if English was the national language of communication. There were no exclusion criteria.

### Data collection tool

Data was collected through a questionnaire developed by the study team. The items in the questionnaire were drawn from the review of literature on oral health policies, universal health coverage, and types of services provided through primary health care (22–27). The development of the questionnaire (questions) was done through an iterative process among the five team members using the Delphi method. The Dephi method is a consensus-building method employed to ensure unbiased input and minimizing the impact of social desirability or power dynamics (28). The team lead (BG) developed the initial questionnaire which was distributed to team members to rate the relevance questionnaire items to the overall research questions and offer comments. Ratings and comments were used to make modifications to the questionnaire. The updated version of the questionnaire underwent another round of rating and commenting in an iterative process that persisted until a consensus was achieved.

Thereafter, the revised questionnaire was reviewed and finalized by ten experts in the fields of dental public health and pediatric dentistry. The experts were drawn from the ECC Advocacy Group (https://eccagroup.org/) which included experts working on ECC from 53 countries. The aim was to engage experts from different regions to enhance the generalizability of the data collection tool. The call for participation in the expert review of the questionnaire was made on the listserv of the group.

The survey tool included nine questions on the countries' background information. These questions inquired about the FDI and/or IAPD membership status of participating countries, presence of oral health directorate or an assigned dental or oral health officer in the country, and the presence of a national oral health policy document, position statement on oral health, and/or an oral health policy as part of the general health policy. A tick of the checkbox corresponding to any of the questions was an affirmative response. Multiple responses were possible.

The survey also asked the question: which of the following is/are addressed by your country oral health policy/ies? The question had 11 response options that inquired about sugar consumption reduction strategies, oral health promotion, oral health education, interprofessional collaboration, and oral health surveillance. A tick of the checkbox corresponding to any of the questions was an affirmative response. Multiple responses were possible.

The questionnaire also inquired about whether dental or oral health care was a part of universal health coverage in their country. Responses were yes or no. If yes, they were asked if it was partial or complete coverage.

The last question was an open-ended question that encouraged participants to provide additional details on their country's national policies and/or share any relevant comments. See Supplementary Table S1 for the study questionnaire.

### Study procedure

An invitation explaining the objective of the survey was drafted by the research team and posted along with the questionnaire on the FDI website under the “survey” section between December 2020 and December 2021. The Chief Dental Officer of Canada assisted the research team with informing his global counterparts from other FDI member nations about the survey. For countries with no responses, the study team searched recent pediatric oral health literature and directly reached out to corresponding authors who were experts in the field. Authors of recently published data, such as those who were affiliated with the Ministry of Health in their respective country, were contacted through emails provided in the published papers. In addition to explaining the purpose of the study, these experts were encouraged to either contact their chief dental officer or complete the survey questionnaire.

### Statistical analysis

Statistical analysis of the quantitative variables was descriptive (frequencies and percentages). Thematic analysis was applied for the responses to the open-ended questions, which were first reviewed for similar themes: the frequency of responses referencing a given thematic element determined the themes. One researcher (BG**)** reviewed and arranged all open-ended responses. In those instances where more than one participant responded for a particular country, the responses were pooled and presented collectively for each country. Responses were categorized by continent and within continent by country.

### Ethical considerations

This study was approved by the deanship of scientific research—Imam Abdulrahman bin Faisal University (IRB-2023-02-029). Participants indicated their consent to participate in the study by ticking a check box. Respondents who checked the box were able to go on to complete the survey. An introductory section preceded the questionnaire and debriefed participants about the study purpose and data collection, as well as assured participants of the confidentiality of their responses and their voluntary participation.

## Results

A total of 442 individuals viewed the survey invitation, 217 responded to the survey, and 87 surveys had complete responses. The 217 partial or fully completed surveys represented data from 60 out of the 158 FDI member-countries, yielding a country level participation rate of 38%.

Table 1 provides an overview of the status of the 60 countries that participated in the survey. A total of 54 (62.1%) of the 60 countries had an oral health directorate/division within their National Ministry of Health and 48 (55.2%) had an oral health policy or position statement on oral health.

**Table 1:** Background information about the participating countries.

Items	Response/country (%)		
	Yes	No	Do not know
Is your country a World Dental Federation (FDI) member?	58 (66.7)	4 (4.6)	25 (28.7)
Is your country a member of International Association of Pediatric Dentistry (IAPD)?	46 (52.9)	13 (14.9)	25 (28.7)
Does your country have an oral health Directorate/Division in the National Ministry of Health?	54 (62.1)	21 (24.1)	8 (9.2)
Does your country have a national oral health policy document OR position statement on oral health?	48 (55.2)	21 (24.1)	17 (19.5)
Does your country have an oral health recommendation/statement as a part of a general health policy document?	48 (55.2)	16 (18.4)	22 (25.3)
Does your country have general health policy that addresses issues that are relevant to oral health (national tax on sugars, obligatory dental check-up within pediatric visits)?	47 (54.0)	28 (32.2)	11 (12.6)
Does your country have a National Universal Healthcare (UHC) plan?	54 (62.1)	15 (17.2)	17 (19.5)
If your country has a National Universal Healthcare (UHC) plan does it include dental care?	42 (48.3)	14 (16.1)	27 (31.0)
Does your country have specific programs that target the oral health care needs of socially disadvantaged child populations?	30 (49.4)	25 (28.7)	18 (20.7)

Further, 48 (55.2%) of the 60 countries had oral health covered within their general health policies, 47 (54%) had general health policies that were relevant to oral health (e.g., sugar taxation, obligatory dental checkups within pediatric visits); 30 (49.4%) countries had programs targeting the oral health care needs of socially disadvantaged populations.

In addition, 35 (57.5%) countries had oral health promotion programs run by dental professionals of which 40 (66.7%) were members of FDI and 32 (52.9%) were members of International Association of Pediatric Dentistry (IAPD). Furthermore, 54 (62.1) countries had a National Universal Health care (UHC) plan and 42 (48.3) have dental care included in their UHC.

**Figure 1:**
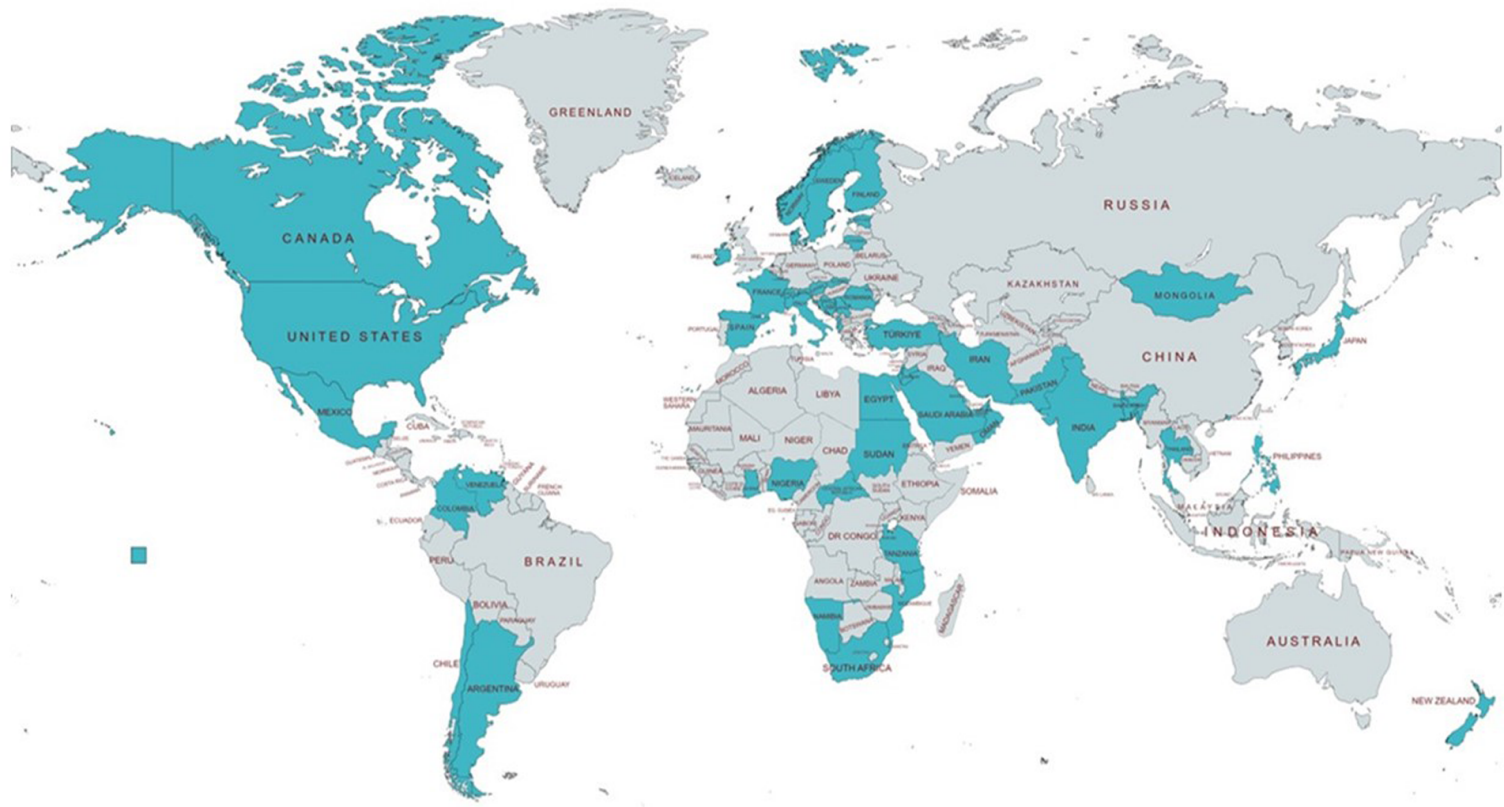
Countries included in the study are colored (*N* = 60).

Figure 1 is a pictograph of the countries that participated in the study. There were ten from Africa, eight from North America, four from South America, twenty-one from Europe, sixteen from the Asia, one from Oceania and no response from Australia.

**Figure 2:**
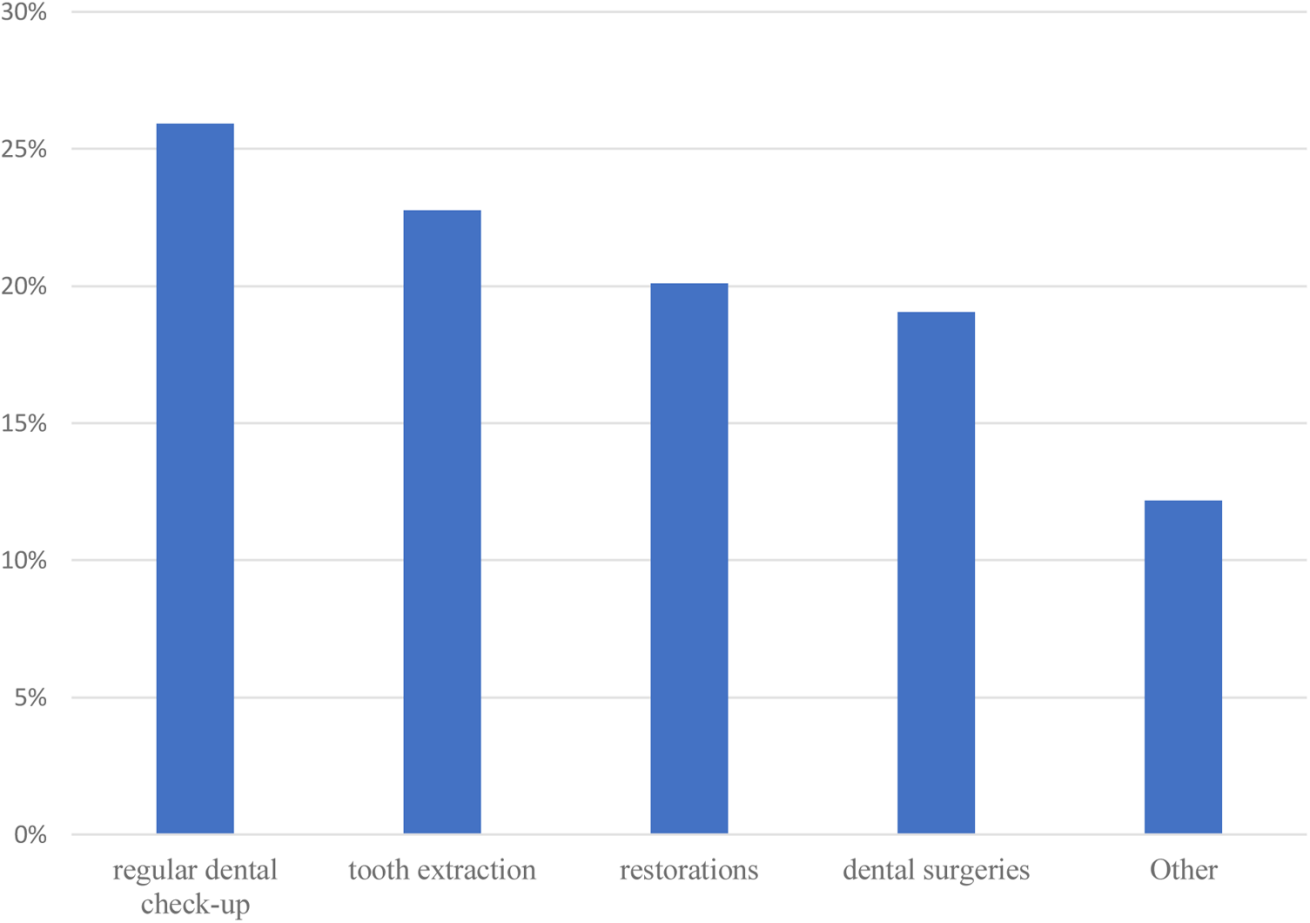
Types of dental services covered by universal health coverage plans.

Figure 2 shows the types of dental services covered by countries with UHC. Regular dental checkups were the most common type of services provided (56.3%), followed by extractions (49.4%), restorations (43.7%), and dental surgical procedures (41.4%). Only four (6.9%) countries include oral health care for infants, toddlers, and preschool children as part of their UHC plan.

As shown in Table 2, nine (15%) countries have an oral health policy on sugar taxation. The most common policy thrusts in the national oral health policies were promoting oral hygiene (71.7% of countries), the provision of fluoride products for children (53.3%), collaborating with primary care providers (53.3%), and prenatal oral health education (50.0%).

**Table 2:** Percentages of items included within oral health policy/country.

Which of the following is/are addressed by your country oral health policy/ies? Check all that apply	No (Percentage)
Reduction of sugar consumption	28 (46.7)
Sugar taxation	9 (15.0)
Promotion of oral hygiene measures (brushing days within schools, distribution of toothpastes and toothbrushes, public campaigns)	43 (71.7)
Provision of other fluoride products (fluoride varnish) for children at risk for caries	32 (53.3)
Promotion of first dental visit by 12 months of age	22 (36.7)
Professional collaboration with primary care professionals to provide oral health education/promotion/screening as part of overall child health assessments	32 (53.3)
Coordination with medical providers to facilitate dental counselling, dental screening, and preventive procedures to infants	21 (35.0)
Prenatal mother oral health education	30 (50.0)
Access to oral health care for at risk populations of children (special needs and minorities) as part of early childhood oral health promotion	28 (46.7)
Incorporate individual risk assessment and self-management goals as part of diagnosis and treatment planning	21 (35.0)
Government surveillance systems on oral health	27 (45.0)
Government surveillance systems on dental caries in the primary dentition of infants and preschoolers (0–5 years)	23 (38.3)

Supplementary Appendix Figures S1A–lL are pictographs of the countries reporting on each of the policy thrusts highlighted in this analysis. The figures provide a comprehensive overview of oral health policies across various countries, highlighting key initiatives and geographical distribution. The figures showed that Europe and Asia are prominent regions in implementing policies promoting oral hygiene, sugar reduction, and access to dental care. Specifically, Europe leads in policies related to sugar reduction, oral hygiene measures, fluoride provision, and early dental visits, while Asia leads in professional collaboration and coordination with medical providers. Africa, North America, and Oceania also demonstrate significant engagement in oral health policy implementation, albeit to a lesser extent compared to Europe and Asia. The figures underscore the global efforts to address oral health disparities and promote preventive measures across diverse regions.

### Continental coverage of oral health promotion programs

#### Africa

All ten (17.9%) of the 56 countries in Africa that took the survey have a position statement or an oral health policy and an oral health division/directorate except for Ghana. Eight countries (Egypt, Ghana, Mozambique, Namibia, Nigeria, Senegal, South Africa, Tanzania) have policies that include promotion of oral hygiene measures; four (Egypt, Ghana, South Africa, Sudan) provide fluoride products like fluoride varnish for children at risk for caries; and two (Ghana and Egypt) have policy that promotes first dental visit by 12 months of age. In addition, six countries (Egypt, Ghana, Nigeria, Senegal, Sudan, Tanzania) have policies that promoted professional collaboration with primary care professionals to provide oral health education and screening as part of overall child health assessments. Prenatal mother oral health education was part of oral health policy in Egypt, Ghana, Namibia, Nigeria, Senegal, and Sudan; and two countries (Egypt and Namibia) advocate caries risk assessment for children. Although government surveillance systems on oral health are present in five countries (Egypt, Ghana, Namibia, Sudan, Tanzania), only Egypt, Namibia, and South Africa conduct surveillance of dental caries in 0–5 years old. In addition, Namibia has implemented sugar taxation, Egypt, Ghana, Nigeria, Senegal, and Tanzania have policies on the reduction of sugar consumption, and Egypt, Ghana, Namibia, Senegal, and Sudan have programs for those with special needs and socially disadvantaged populations.

## Asia

Seventeen (including Hong Kong, a territory of China that we referred to as a country for the ease of analysis) (35.4%) of the 48 countries in Asia responded to the survey. Of these, 11 countries (Bahrain, Hong Kong, Iran, India, Israel, Japan, Philippines, Saudi Arabia, Thailand, Turkey, United Arab Emirates) have a directorate for oral health program and a position statement on oral health and two (Bangladesh and Palestine) have a directorate for the oral health program. Twelve countries (Bangladesh, Bahrain, India, Israel, Japan, Jordan, Mongolia, Philippines, Saudi Arabia, Thailand, Turkey, United Arab Emirates) include and address oral health as part of the general health policy.

Twelve countries (Bangladesh, Bahrain, Hong Kong, Iran, Japan, Jordan, Mongolia, Palestine, Philippines, Thailand, Turkey, United Arab Emirates) have policies that promote oral hygiene measures and first dental visits by first year of age; while six of these 12 countries (Hong Kong, Mongolia, Palestine, Jordan, Thailand, Turkey) include oral hygiene measures such as brushing days within schools, distribution of toothpastes and toothbrushes and public oral health promotion campaigns as specific activities in their policy. In addition, 10 countries have oral health surveillance and surveillance system on dental caries for preschoolers (Bahrain, Hong Kong, Iran, Japan, Oman, Palestine, Philippines, Saudi Arabia, Thailand, and United Arab Emirates).

Six countries (Israel, Oman, Philippines, Thailand, Saudi Arabia, and United Arab Emirates) have policies on sugar taxation and reduction of sugar consumption, while Iran has only a policy on reduction of sugar consumption. Twelve countries (Bahrain, Hong Kong, Iran, Israel, Palestine, Oman, Pakistan, Philippines, Saudi Arabia, Thailand, Turkey, and United Arab Emirates) have programs for those with special needs and disadvantaged populations; and eight countries (Bahrain, Hong Kong, Iran, Israel, Philippines, Thailand, Turkey, United Arab Emirates) have policy statements on professional collaboration with primary care professionals to provide oral health education/promotion/screening as part of overall child health assessments, and coordination with medical providers to facilitate dental counselling, dental screening and preventive procedures to infants and prenatal mother oral health education. Israel, Jordan, Palestine, Saudi Arabia, and United Arab Emirates highlighted prenatal mother oral health education as part of the policy requirement on professional collaboration with primary care professionals.

## Europe

Twenty (40%) of the 50 countries in Europe responded to the survey. Five countries (Ireland, Denmark, Serbia, Norway, and Sweden) have a national oral health policy; France has a health policy that addresses oral health; six countries (Czech Republic, Italy, Denmark, Serbia, Macedonia, Romania) have an oral health recommendation/statement as a part of a general health policy; and five countries (Albania, Austria Estonia, Finland, and Lithuania) have a general health policy that addresses issues that are relevant to oral health (national tax on sugars, obligatory dental check-up within pediatric visits). Denmark and Serbia have a directorate of oral health. Seven countries (Croatia, Denmark, Estonia, Finland, Ireland, Spain, and Switzerland) have government surveillance systems, while six of these seven countries (Croatia, Denmark, Estonia, Finland, Ireland, and Switzerland) have surveillance systems for infants and preschoolers. Four countries (Spain, Denmark, Czech Republic, and Ireland) have programs for those with special needs and socially disadvantaged populations. Eight countries (Bosnia, Estonia, Herzegovina, Lithuania, Romania, Serbia, Slovakia, Spain, and Switzerland) promote oral hygiene measures, and eight countries (Croatia, Estonia, Finland, Lithuania, Romania, Slovakia, Spain, and Switzerland) provide fluoride products for children at risk for caries. Prenatal oral health and dental homes were part of oral health policies in only two countries (Denmark and Romania). Croatia, France, Estonia, Finland, Romania, Spain, and Slovakia have a policy on reduction of sugar consumption, while Denmark has a policy on both the reduction of sugar consumption and sugar taxation.

## North America

Eight (34.8%) of the 23 countries in North America responded to the survey. Seven countries (Antigua, Barbuda, Canada, Mexico, Puerto Rico, Trinidad and Tobago, and the United States) have a directorate of oral health, four countries (Canada, Mexico, Trinidad and Tobago and the United States) have a national oral health policy, and four countries (Canada, Montserrat, Puerto Rico, and the United States) also address oral health as a part of a general health policy Four countries (Canada, Mexico, Trinidad and Tobago and the United States) have a policy on reduction of sugar consumption; Mexico has a policy on sugar taxation.

Five countries (Mexico, Montserrat, Puerto Rico, Trinidad and Tobago and the United States) have policy that promotes oral hygiene measures (brushing days within schools, distribution of toothpastes and toothbrushes, public campaigns); three countries (Mexico, Puerto Rico, and the United States) provide fluoride varnish for children to reduce the risk for caries; the Unites States promotes first dental visit by 12 months of age; and three countries (Montserrat, Trinidad and Tobago and the United States) include professional collaboration with primary care professionals to provide oral health education/promotion/screening as part of overall child health assessments. In addition, four countries (Trinidad and Tobago, Montserrat, Puerto Rico, and the United States) have a policy on coordination with medical providers to facilitate dental counselling, dental screening and preventive procedures to infants including prenatal maternal oral health education.

Three countries (Puerto Rico, Trinidad and Tobago and the United States) provide access to early childhood oral health promotion for at-risk populations of children (special needs and minorities) while the United States has oral health programs for all those with special needs. Mexico, Trinidad and Tobago and the United States have government surveillance systems on oral health, with only Trinidad and Tobago and the United States including surveillance systems for ECC.

## South America

Four (33.3%) of the 12 countries in South America responded to the survey. Argentina, Chile and Columbia have a national oral health policy, an oral health statement as a part of a general health policy and a general health policy that addresses issues that are relevant to oral health. Venezuela has an oral health directorate in the National Ministry of Health. Only Colombia has a policy that promotes oral hygiene measures (brushing days within schools, distribution of toothpastes and toothbrushes, public campaigns) as well as the provision of fluoride varnish for children at risk for caries. Programs for those with special needs are part of oral health policy in Argentina.

## Oceania

Of the 14 Oceania countries, only New Zealand provided a response to the survey. The country has an oral health directorate in the National Ministry of Health, a national oral health policy document and an oral health statement as a part of a general health policy document. There are national oral health promotion programs aimed at preschoolers and low-income families, and policies on reduction of sugar consumption, promotion of oral hygiene measures, provision of other fluoride products (such as fluoride varnish) for children at risk for caries and promotion of dental visits by 12 months of age. In addition, its oral health policy includes professional collaboration with primary care professionals to provide oral health education/promotion/screening as part of overall child health assessments. The country also has a government surveillance system on oral health and surveillance systems for children 0–5 years.

### Current status on universal health coverage

The comprehensive oral health programs implemented through universal health coverage schemes includes the provision of regular dental check-ups, tooth extraction, restorations, and dental surgeries (22). Of the nine countries in Africa that participated in the survey, Senegal and Nigeria have health insurance schemes that cover regular dental check-up, tooth extraction, restorations and include complete oral health care for infants, toddlers, and preschool children.

In Asia, six countries (Jordan, Israel, Oman, Pakistan, Thailand, and UAE) have comprehensive UHC packages that include toddlers and preschoolers, two countries have comprehensive UHC oral health programs that do not include infants, toddlers and preschoolers (Hong Kong and Philippines), and two countries (Japan and Turkey) have partial UHC programs that include toddlers and preschoolers. In Iran the program covers only tooth extractions and dental restorations and partially covers toddlers and preschoolers while the comprehensive UHC program in Bahrain covers children and includes orthodontic treatment. The Bangladesh UHC program does not include dentalcare.

In Europe, five countries (Czech Republic, Denmark, Estonia, Finland, Ireland, Norway) have comprehensive UHC that includes children below 6 years; and in six countries (Austria, France, Macedonia, Spain, Albania, and Italy) UHC is partial and includes infants, toddlers, and preschoolers. Sweden provides comprehensive UHC for children up to the age of 24 months; Lithuania and Serbia have comprehensive UHC for children from 0 to 8 years; and five countries (Czech Republic, Denmark, Estonia, Finland, Ireland, Norway) have programs that provide different services for different age groups. Spain has a program that covers restorations in the permanent dentition of children 6–16 years old. The Czech Republic not only provides comprehensive UHC but also provides prosthodontic and orthodontic care for adults.

In North America, Mexico and Trinidad and Tobag, comprehensive UHC includes oral health care for infants, toddlers, and preschool children. The coverage extends to children up to age 18 in Trinidad and Tobago. In Montserrat there is no UHC, but governmental dental service is entirely free to specific groups of persons in the community including children, adolescents and young people receiving tertiary education. Puerto Rico has a health reform program that provides regular dental check-ups, fluoride varnish application (every 6 months), restorations, dental surgery, and sealants for the Medicaid and Medicare-eligible, and the medically indigent (federal poverty level below 200%) populations through the Government Health Insurance coverage.

In South America, Chile has partial UHC that includes oral health care for infants, toddlers and preschool children and covers only regular dental checkups. Colombia has UHC which covers regular dental check-up, tooth extraction and restorations.

In Oceania, New Zealand has a comprehensive UHC that includes oral health care for infants, toddlers and preschool children and covers free basic dental care up to 18 years old.

**Figure 3:**
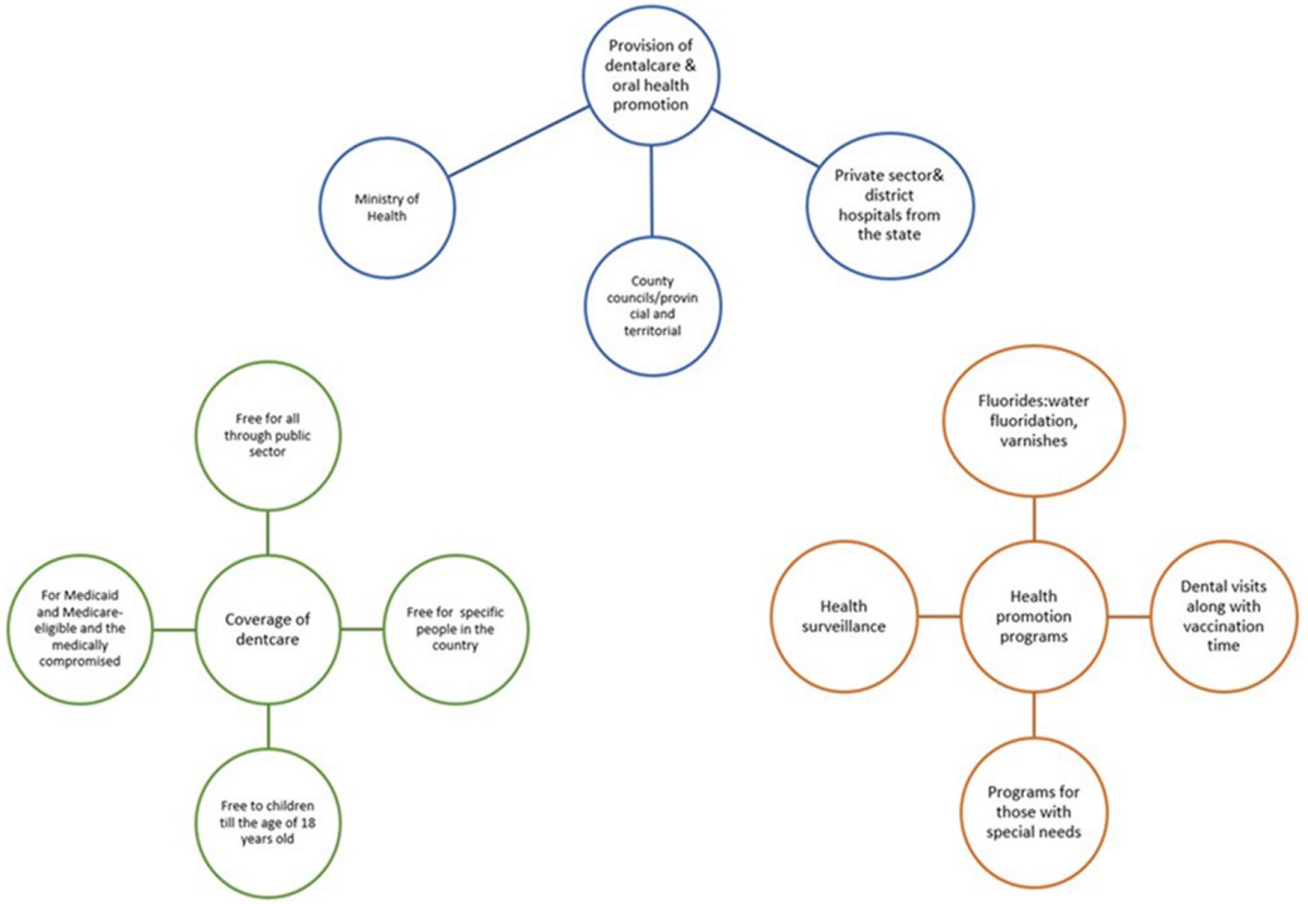
Summary of the comments by respondents.

Figure 3 provides a thematic analysis of the open-ended question(s). The main themes identified were how dental care was provided, the coverage of dental care and the type of health promotion programs.

## Discussion

This study provides the first publicly accessible mapping of national oral health policies regarding children and is also the first study to investigate the extent and existence of oral health policies highlighting the variations between countries with different health service delivery models. The study findings suggest that the status of oral policies differs by continent and with continents. For example, there is variation in the ways oral health programs are mapped against the general health policy, implemented across different age spectrums, and the extent and coverage of oral health care by the government. In addition, countries in Europe and North America have policies that address the oral health needs of diverse populations including those with special needs. Varying status of national oral health policies were reported ranging from complete absence of a policy to well-developed oral health policies to partial, complete and services beyond the basic requirements for oral health coverage to ensuring young children and those with special needs had access to oral care.

The study findings indicate that not all countries have well-defined oral health policies. Oral health policy formulation and implementation is a complex process, and countries may develop oral health polices for different reasons ranging from rational comprehensive thinking, wherein the cost of any policy exceeds its benefits (24, 25, 29), to the use of an incremental approach to policy formulation wherein the focus is on the ultimate benefit accrued over time and even when only the minority benefits (30). Policy development may also arise from public demand from the broader public or an elite group (31) or may be influenced by the type of health system the nation operates, its purchasing power, the impact of the private sector, and the extent of international influence on the health system (32). Whatever the reasons and the process for policy formulation, policies help improve practices for the collective (33). The presence of oral health policies in less than half of the countries surveyed raises the need to advocate for bodies, such as the FDI and IAPD, to help support member countries to formulate and/or update national position statement/oral health policies that can drive oral health service provisions for the diverse population.

Also of importance is the inclusion of oral health into general health policies. Slightly more than half (55.2%) of the countries that participated in this survey had oral health included in the general health policy. Oral diseases have a direct impact on overall health and quality of life (34), thus using a common risk approach to guide and plan for health promotion and disease prevention through the creation of an integrated health system can result in enhanced general health and wellbeing in a cost-effective manner (27). Integration at the Primary Health Care (PHC) is feasible and extremely helpful (24), especially for low-income countries with limited resources, limited capacity to expand dental services, and limited dental taskforces (35). Going forward, countries that do not have oral health policies but do have general health policies can be supported to mainstream oral health context into the existing general health policies to maximize the existing resources to facilitate oral health care while mobilizing for new resources to create systems and structures for oral health care.

Importantly, the inclusion of oral health care within the UHC program as a means of promoting access to a range of oral health services with minimal out-of-pocket expenditure for populations is important (36). Eliminating financial hardship while accessing oral health care can facilitate uptake and use of preventive care. While many countries have adopted UHC to reduce healthcare disparities, several counties have not included oral healthcare in the UHC. The autonomy of states and jurisdictions as reflected in the case of France may introduce a layer of complexity with defining country level systems and structures for implementing a unified UHC program. For instance, Saudi Arabia and Italy provide all citizens and residents healthcare mainly through the public sector with some private or private/public entities (37). Ireland provides oral healthcare through a hybrid model with a private/public mix of service provision, predominantly organized based on fee-per-item remuneration (38).

These context specific adaptations of systems and structures to the global call for UHC are important. However, the absence of a global evaluation of these models to access their contributions to the control of oral diseases creates a gap in maximizing the opportunities created by the global call to improve oral health responses at the country level in all countries of the world. The FDI is in a position to conduct this mapping and make this call among its member states in an effort to drive its policy agenda of reducing sugar consumption by adults and children (39), and enforcing sugar taxation (40), school-based oral disease interventions (41, 42) and integration of oral health into maternal and child health programs (43). Increased sugar consumption is linked to dental caries as well as many other medical conditions such as obesity and diabetes (40). Measures to lower the consumption of sugar, including sugar taxation, are growing in high income countries which witnessed an improvement in the health of their individuals (40). Hower it is unknown if this improvement in the overall oral and general health is the direct impact of policies on sugar reduction or the social determinants of health in these countries. The current study indicates that about 50.6% of responding countries include prenatal mother oral health education as part of their oral health policy. In view of the high success reported about this intervention in high- and low-income countries (44–49), these advocacy efforts can drive these interventions as part of the oral health programs in respective countries.

The current study has some limitations. First, the low response rate and lack of representation of many countries did not allow for a full picture of policy status on a global level. Reminders sent through emails as well as reaching out for key persons in oral health were some attempts to increase the response rate. Second, during the long duration of the study, some countries might have introduced some changes to their policies. For instance, Canada introduced the interim Canada Dental Benefit for children <12 years of age in 2022 and is now launching its Canadian Dental Care Plan in 2024 that will provide dental benefits for uninsured children up to 18 years of age in order to improve access to care (50, 51). Third, the survey was structured for countries that have federal delivery of oral health care, while in some countries health care delivery is the responsibility of provincial and territorial governments. Thus, the responses of some federal officials to the questions will not reflect all of the elements or oral health policy and programming that exist in those countries. Lastly, the survey was conducted in English only, which may have limited the participation from non-English speaking countries.

On the other hand, the use of the FDI platform and the fact that most of the countries which did not respond may not have a comprehensive oral health policy for children, thus not giving an accurate picture of the current situation and highlighting the need for rapid action plans to improve the dental health among young children.

## Conclusion

This study shed light on the global landscape of oral health initiatives, highlighting substantial variations in policy status among continents, from complete, absence to well-developed policies. This underscores the need for concerted efforts to support countries in establishing or updating their national oral health policies that can guide oral health service provisions for diverse populations, including children, using an integrated approach especially at the PHC level. While many countries have adopted UHC to reduce healthcare disparities, not all have incorporated oral healthcare into their UHC programs. The context-specific adaptations of healthcare systems and structures to the global call for UHC emphasize the need for a global evaluation of these models to optimize oral health responses worldwide. There is a need for collaborative efforts, advocacy, and global evaluation to drive effective policies and interventions that can enhance oral health on a global scale.

## Data Availability

The raw data supporting the conclusions of this article will be made available by the authors, without undue reservation.
