# Supplementary material for: A global survey of national oral health policies and its coverage for young children
Source: Front Oral Health. 2024 Apr 5;5:1362647. doi: 10.3389/froh.2024.1362647 (PMC11026553; doi:10.3389/froh.2024.1362647)
Supplement: Supplementary file 1 [file Datasheet1.docx]

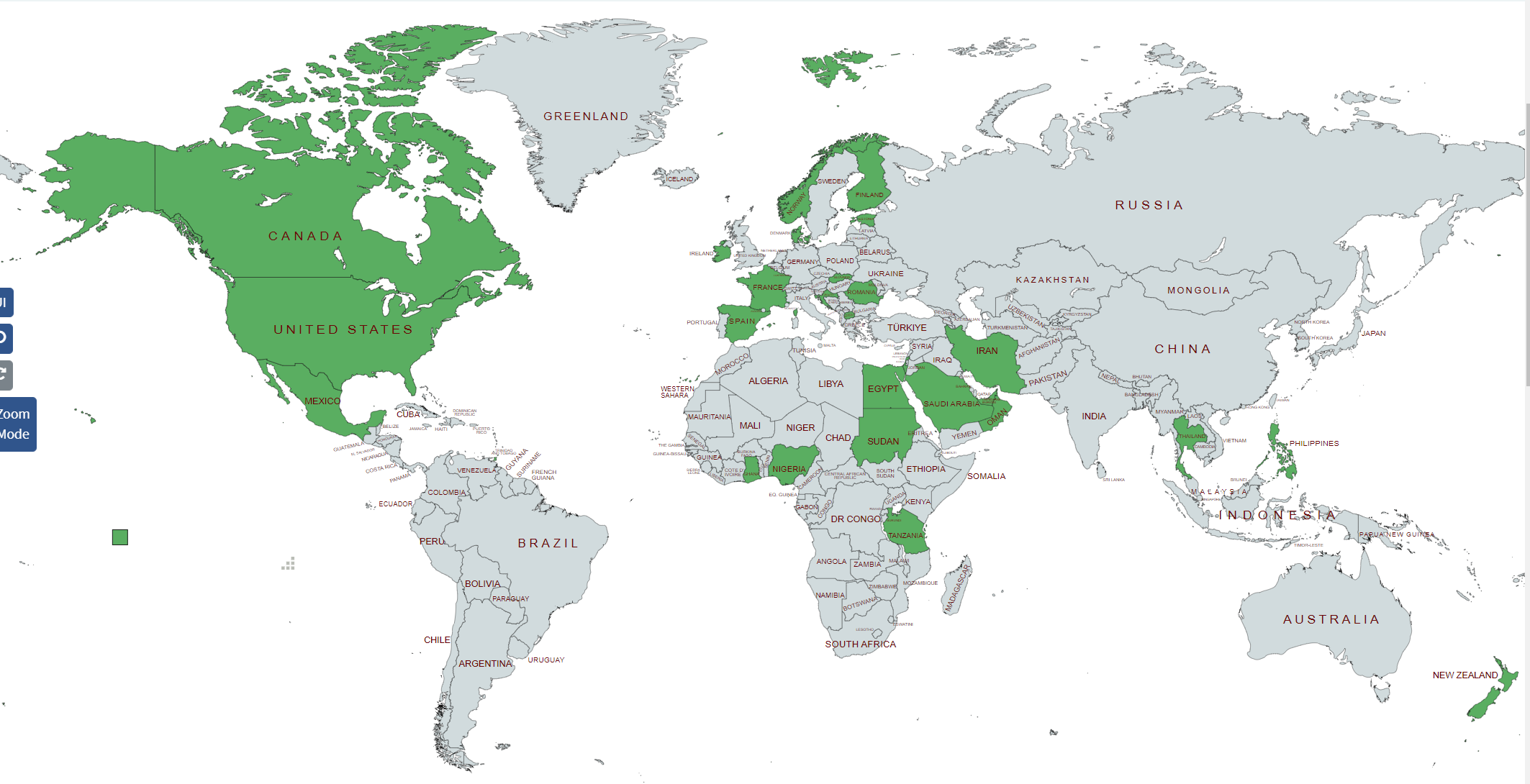


**Appendix Figure 1a: Countries with oral health policy on sugar reduction (N=28)** **The figure shows that most of the 28 countries with oral health policy that promote the reduction of sugar consumption are in Europe.**


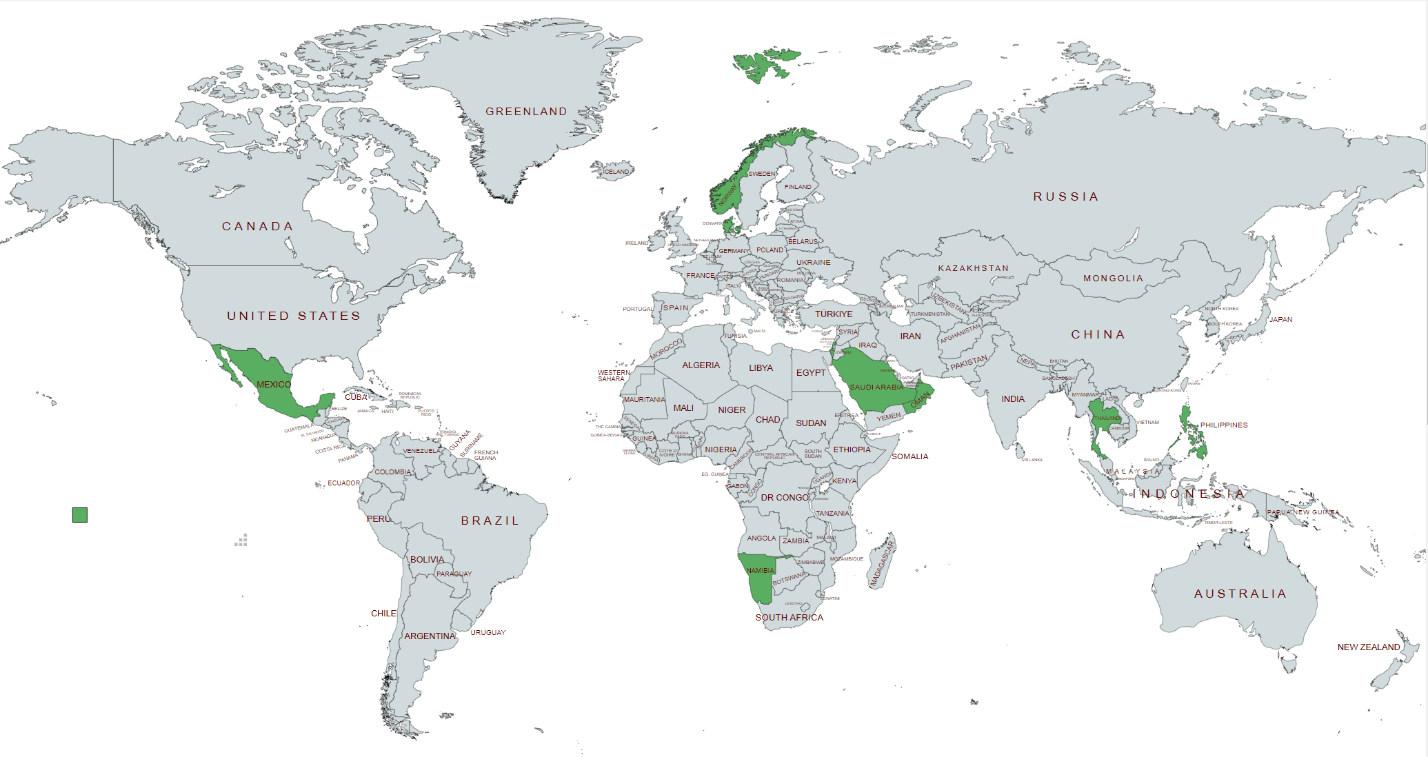


**Appendix Figure 1b: Countries with sugar taxation (N=9). The figure shows that the nine countries with sugar taxation were in Europe and Asia.**


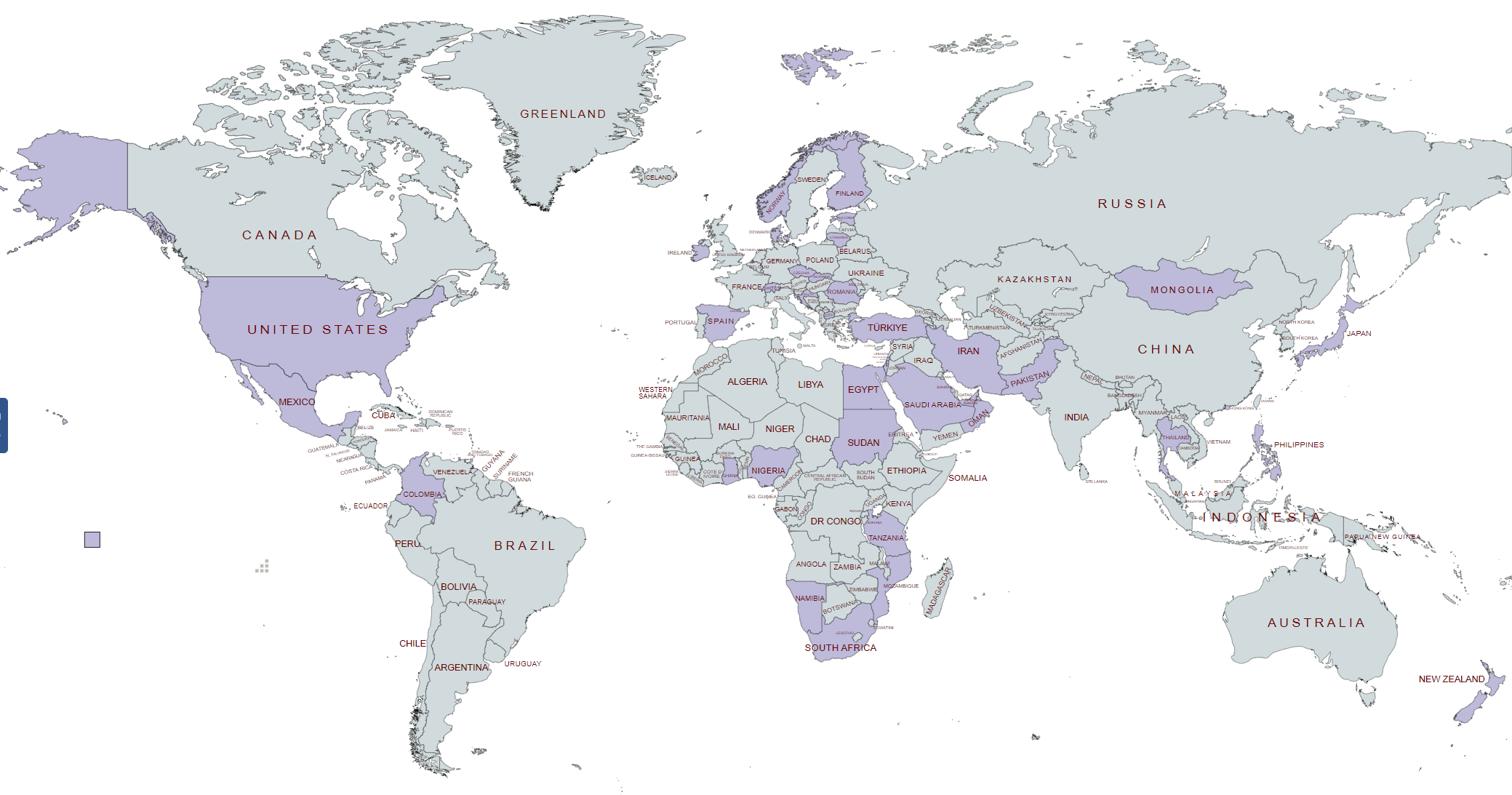


**Appendix Figure 1c: Countries with oral health policy that promotes oral hygiene measures such brushing days within schools, distribution of toothpastes and toothbrushes, public campaigns (N=43).** **The figure shows that the majority of the 43 countries with oral health policy that promotes oral hygiene measures such brushing days within schools, distribution of toothpastes and toothbrushes and public campaigns were mainly in Europe (15 countries) and Asia (13 countries); while nine were in Africa, five in North America and only one in South America.**


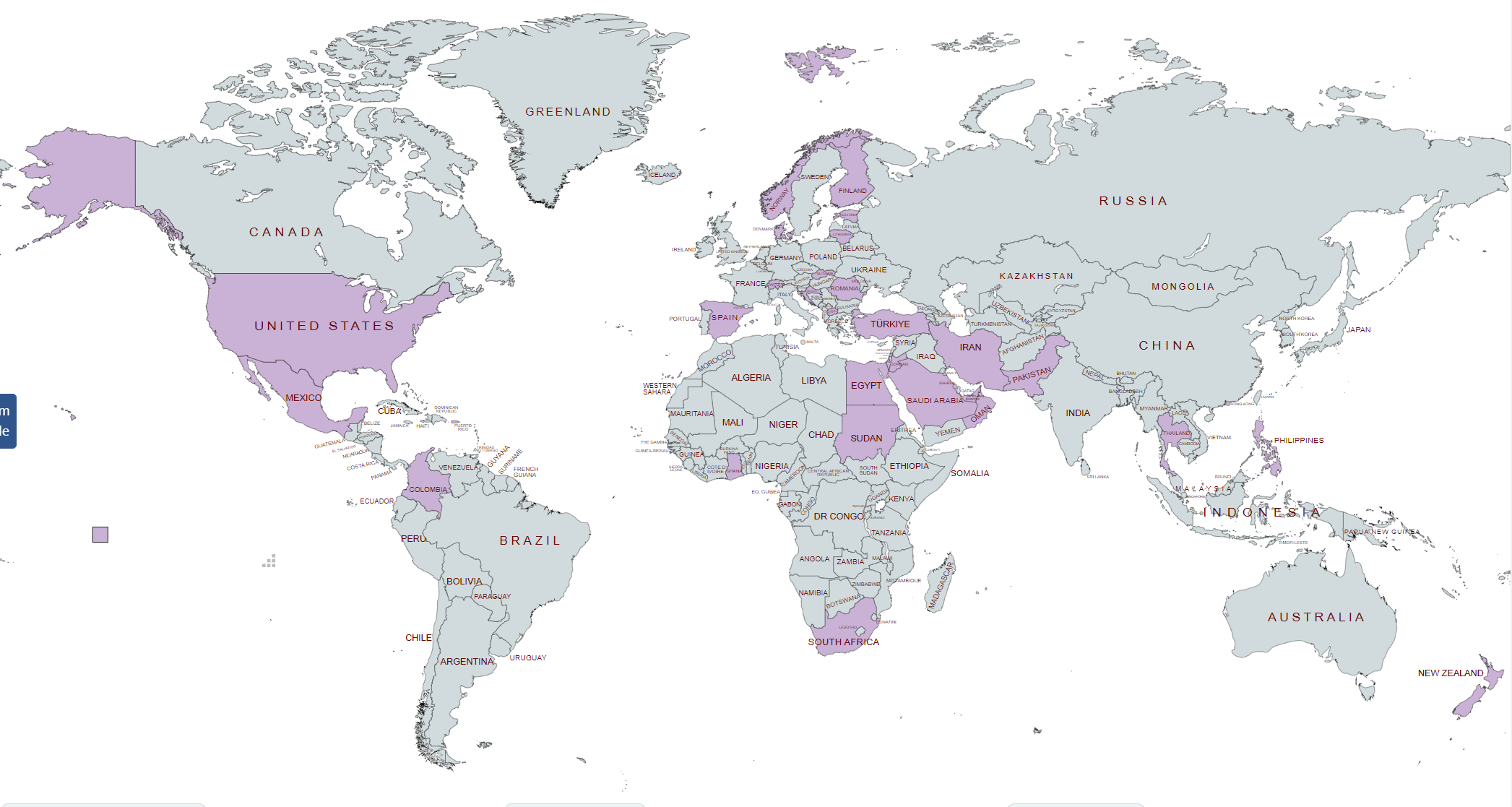


**Appendix Figure 1d: Countries with oral health policy provision of other fluoride products (fluoride varnish) for children at risk for caries (N=32). The figure shows that the 32 countries with oral health policy that includes the provision of other fluoride products (fluoride varnish) for children at risk for caries are located mainly in Europe (12 countries) and Asia (11 countries). While four were in Africa, three from North America and one from South America and Oceania.**


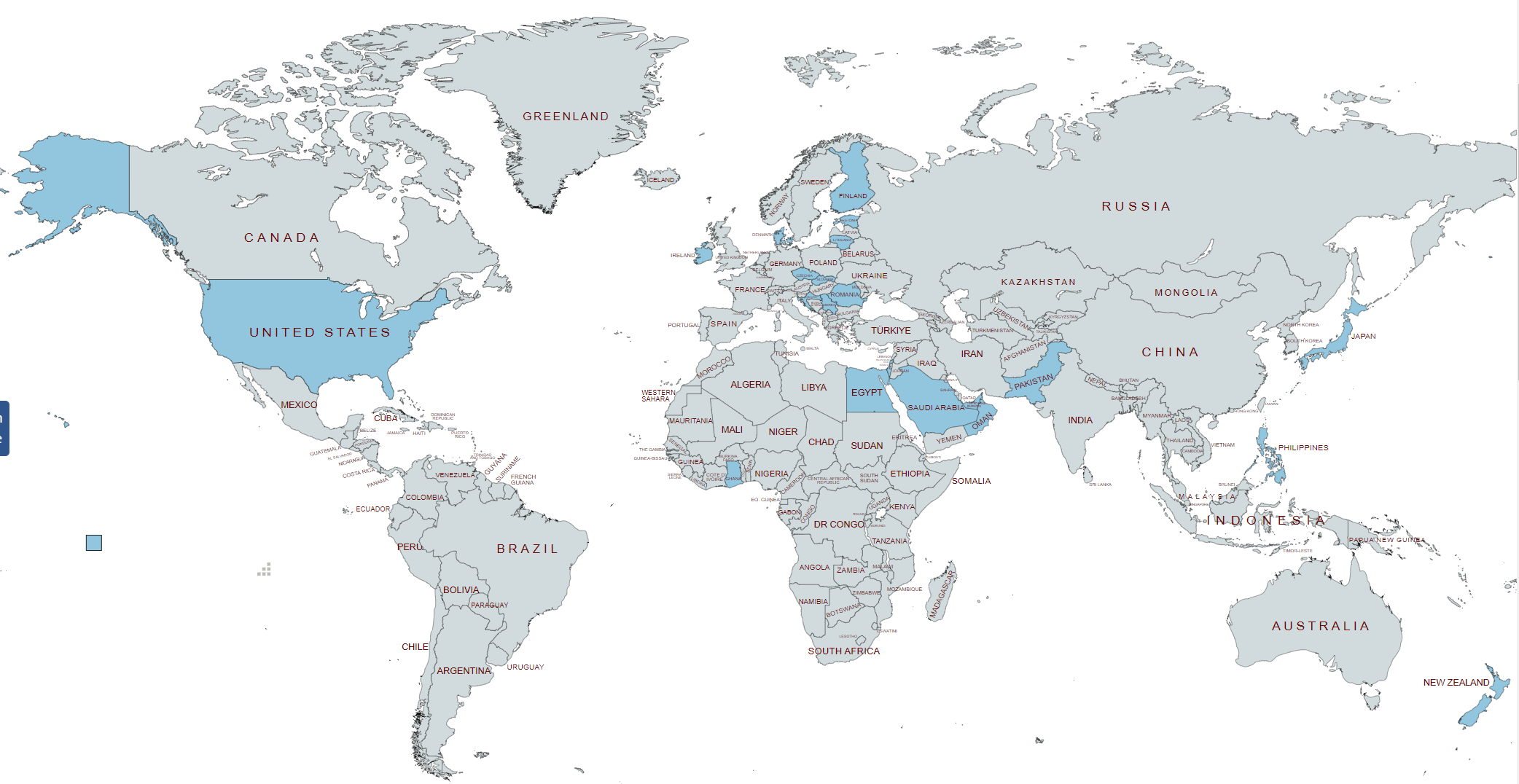


**Appendix Figure 1e: Countries with oral health policy that promote first dental visit by 12 months of age (N=22).** **The figure shows that most of the 22 countries have oral health policy that promotes first dental visit by 12 months of age are located also in Europe (10 countries) and Asia (8 countries) and one from North America and Oceania.**


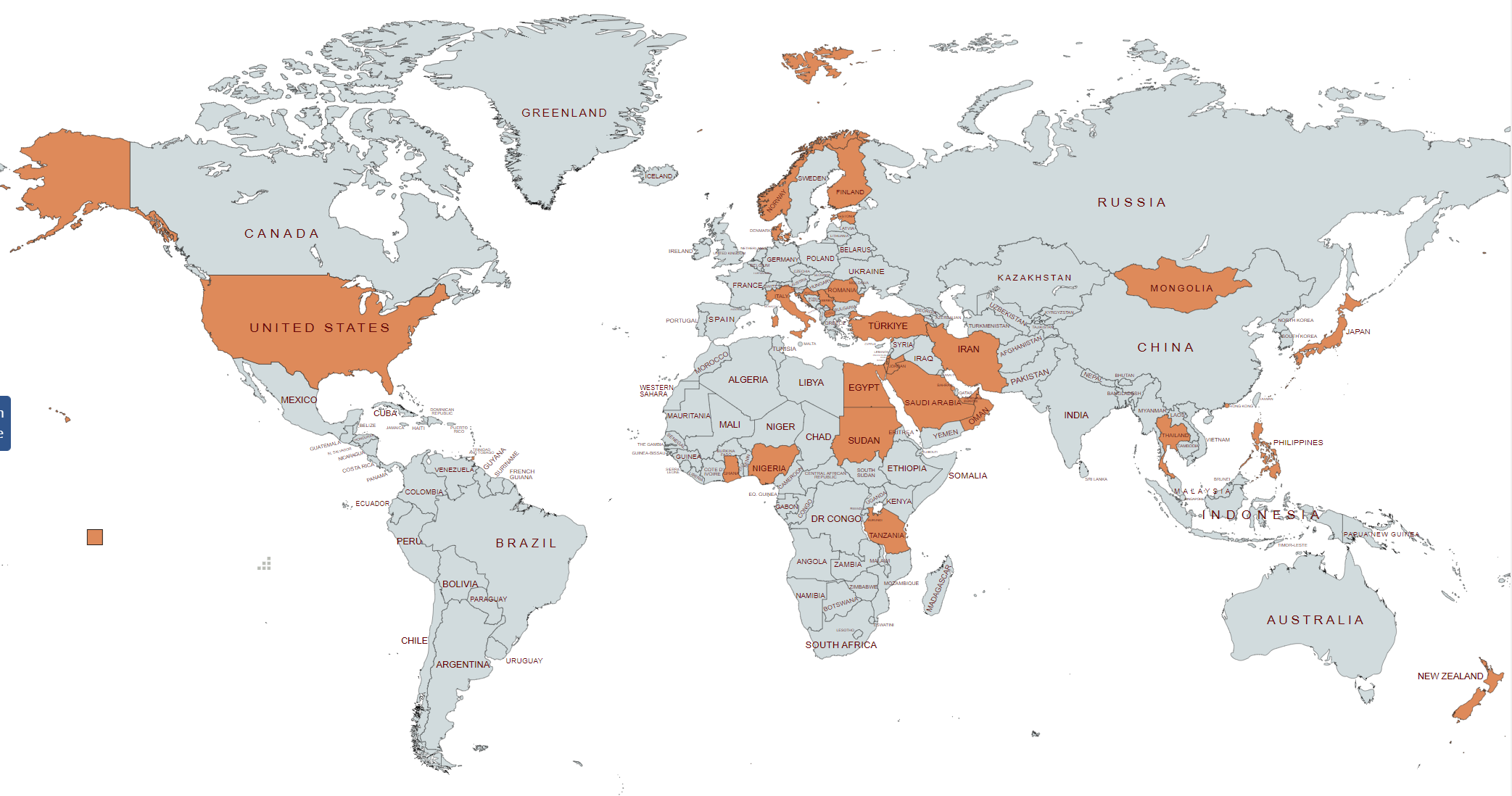


**Appendix Figure 1f: Countries with oral health policy that includes professional collaboration with primary care professionals to provide oral health education/promotion/screening as part of overall child health assessments (N=32). The figure shows that most of the 32 countries with oral health policy that includes professional collaboration with primary care professionals to provide oral health education/promotion/screening as part of overall child health assessments are mainly in Asia (13 countries) and Europe (10 countries). While five countries were in Africa, three in North America and one from Oceania.**


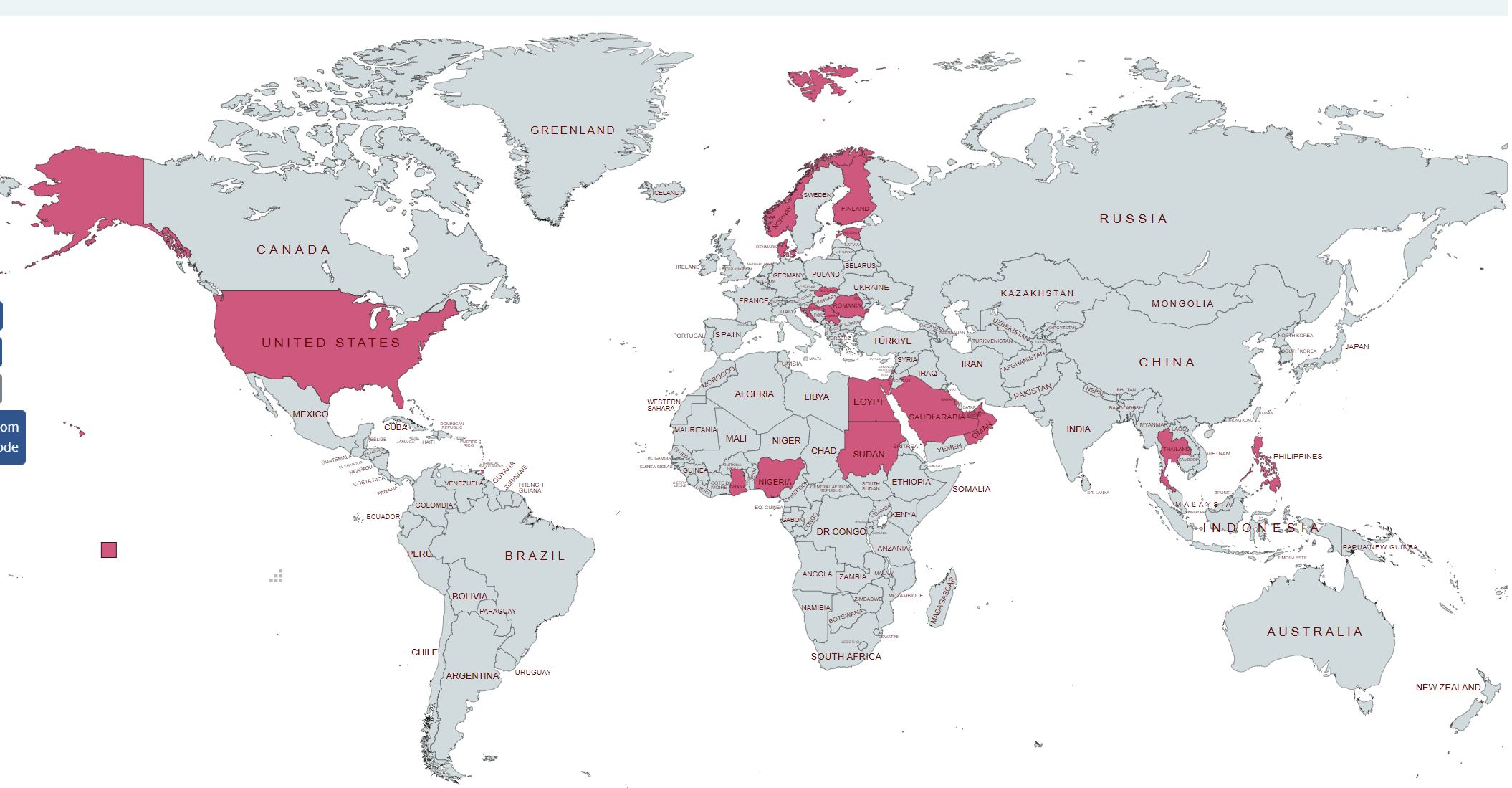


**Appendix Figure 1g: Countries with oral health policy that facilitates coordination with medical providers to facilitate dental counselling, dental screening and preventive procedures to infants (N=21).  The figure shows that the 21 countries with oral health policy that facilitates coordination with medical providers to facilitate dental counselling, dental screening and preventive procedures to infants are located in Europe (8 countries), Asia (7 countries), Africa (4 countries) and two from North America.**


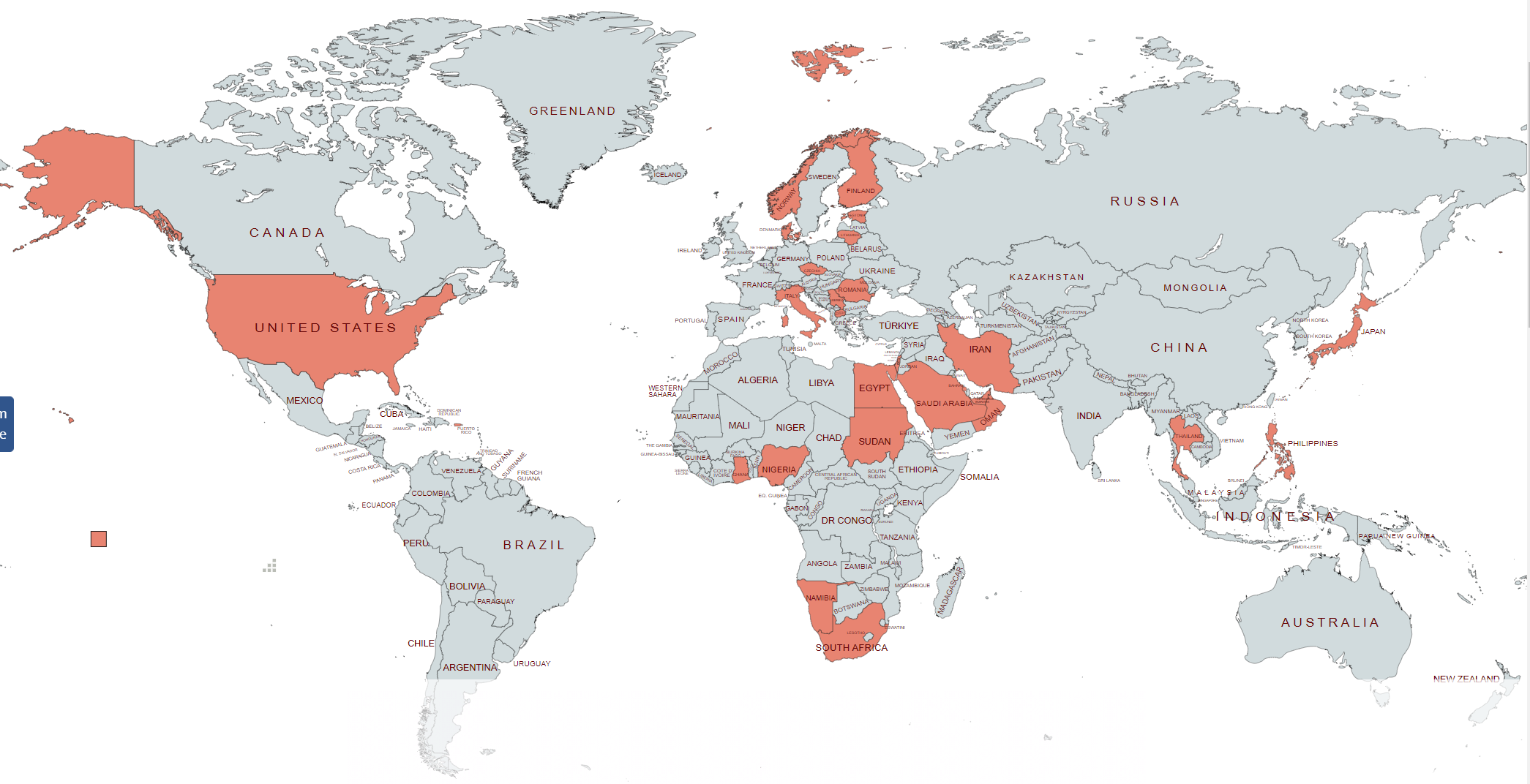


**Appendix Figure 1h: Countries with oral health policy that** **includes prenatal mother oral health education (N=30). The figure shows that the 30 countries with oral health policy that includes prenatal mother oral health education were equally distributed in Asia and Europe (10 countries each) and six were in Africa and four in North America.**


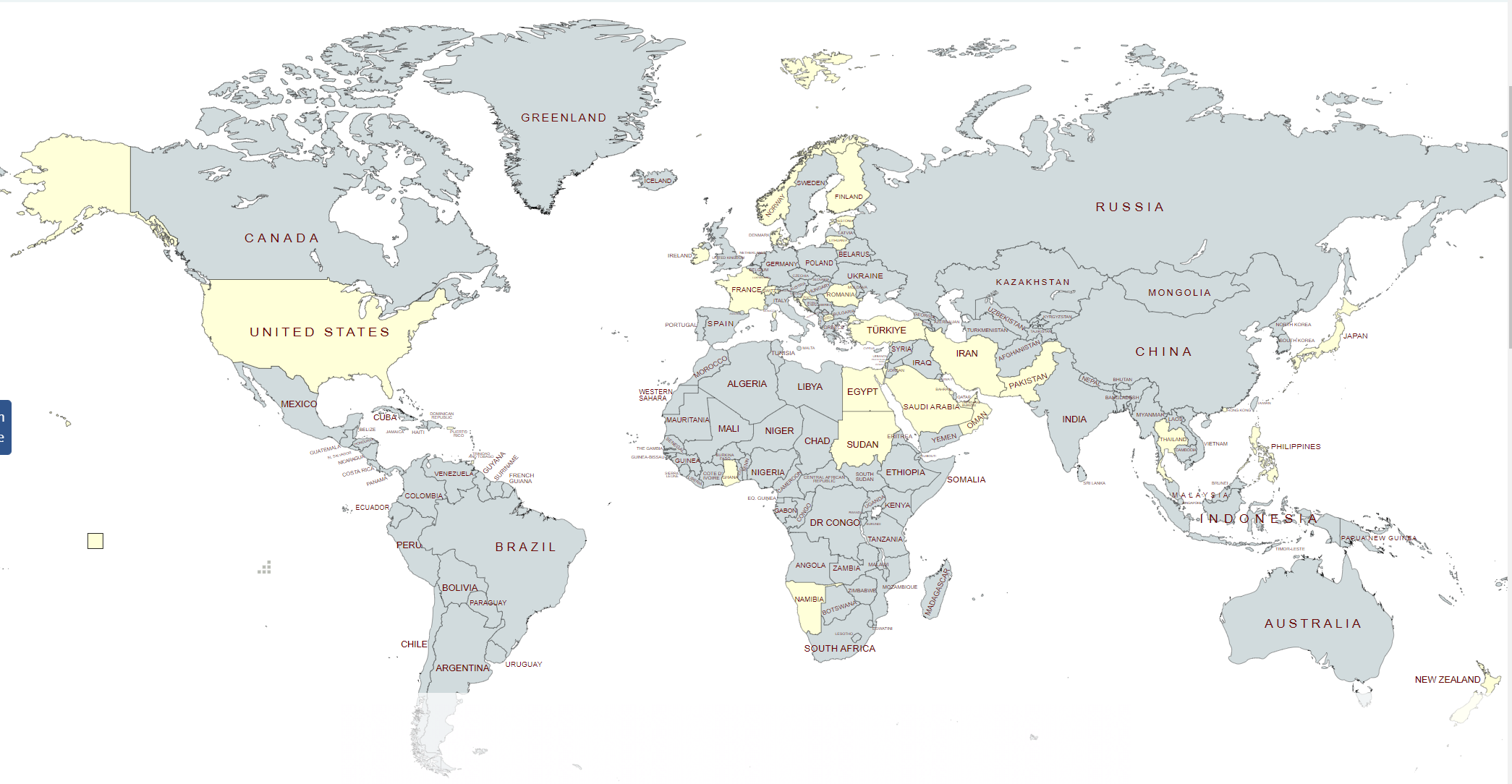


**Appendix Figure 1i: Countries with oral health policy that facilitates access to oral health care for at risk populations of children/special needs and minorities as part of early childhood oral health promotion (N=28).** **The figure shows that the 28 countries with oral health policy that facilitates access to oral health care for at risk populations of children/special needs and minorities as part of early childhood oral health promotion are equally distributed in Asia and Europe (10 countries each) and four were in Africa, three in North America and one in Oceania.**


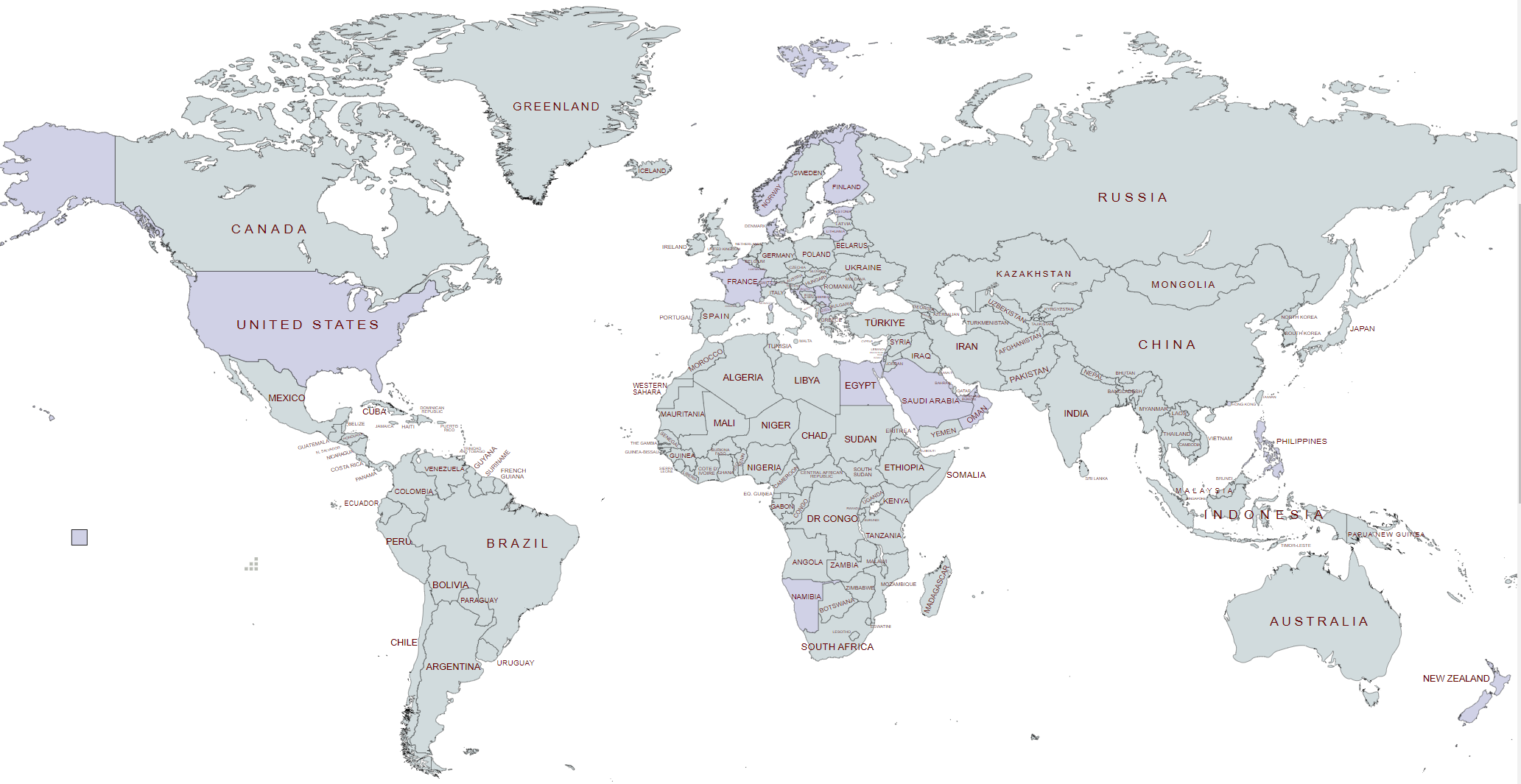


**Appendix Figure 1j: Countries with oral health policy that incorporates individual risk assessment and self-management goals as part of diagnosis and treatment planning (N=21).** **The figure shows that the 21 countries with oral health policy that incorporates individual risk assessment and self-management goals as part of diagnosis and treatment planning are located in Europe (10 countries), Asia (7 countries), two in Africa, and one from North America and Oceania.**


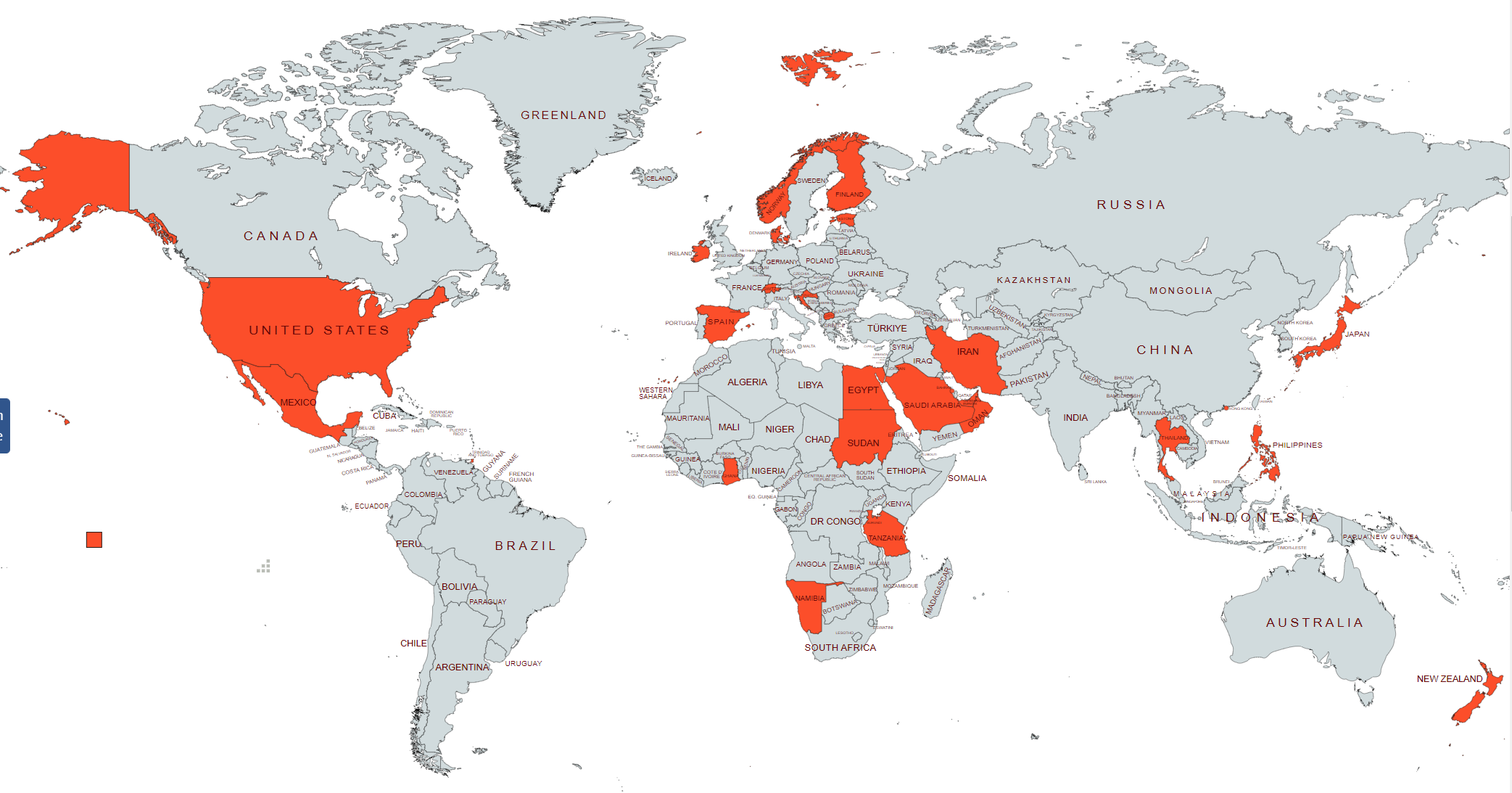


**Appendix Figure 1k: Countries with oral health policy that includes government surveillance systems on oral health (N=27). The figure shows that the 27 countries with oral health policy that includes government surveillance systems on oral health are equally distributed in Asia and Europe (9 countries each) and five were in Africa, three in North America, one in Oceania and none from South America.**


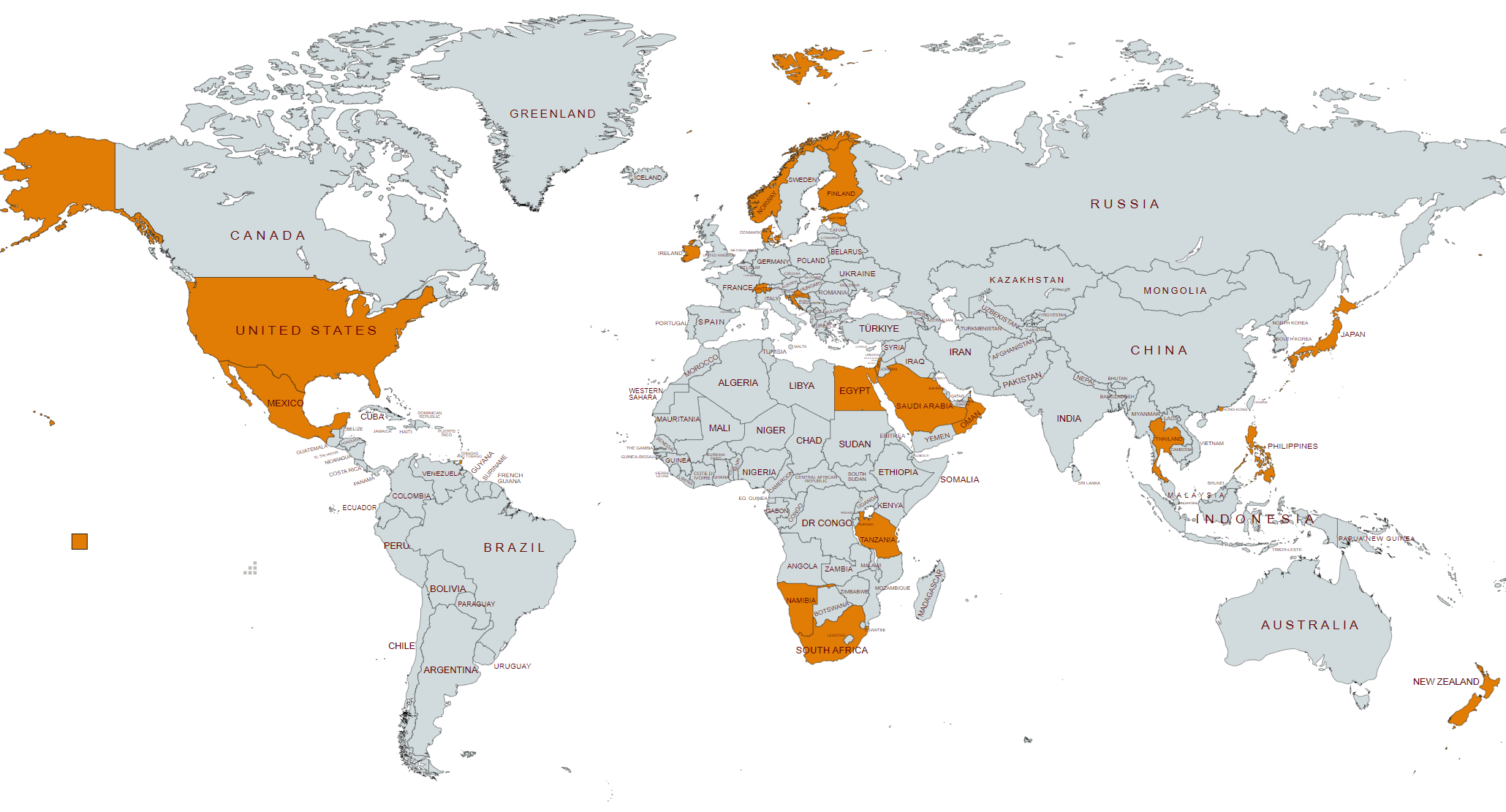


**Appendix Figure 1l: Countries with oral health policy that includes** **government surveillance systems on dental caries in the primary dentition of infants and preschoolers (N=23).  The figure shows the 23 countries with oral health policy that includes government surveillance systems on dental caries in the primary dentition of infants and preschoolers located in Asia (8 countries), Europe (7 countries) and four in Africa, three in North America, one in Oceania and none from South America.**
