# Supplementary material for: A global survey of national oral health policies and its coverage for young children
Source: Front Oral Health. 2024 Apr 5;5:1362647. doi: 10.3389/froh.2024.1362647 (PMC11026553; doi:10.3389/froh.2024.1362647)
Supplement: Supplementary file 2 [file Datasheet2.docx]

**Comments by respondents on their country oral health policy**

| **Country** | **Comment** |
| --- | --- |
| Saudi Arabia | Health promotion is provided mainly by the Ministry of Health (MOH), but other sectors do contribute too. - there’s no UHC in Saudi Arabia. Nonetheless, dental care is available for free at MOH’s health centers and dental schools |
|  | MOH started doing a lot of programs for the preschool children and to promote the dental visit by 12 months with the vaccination time |
| New Zealand | National Oral Health Promotion Program aimed at preschoolers and family, priority populations are Maori (indigenous pop), Pacific and low-income families |
| Republic of Ireland | The Oral Health Policy, Smile ages Slainte is part of the overall UHC package for general health Slanderer. Economists, Sociologists, Health Promotion Academics general public health , dental professionals and general health professionals developed the policy. Ireland is fluoridated so fluoridated programs for children under 6 are treated differently to non-fluoridated countries due to EU legislation . Community programs are to be developed for special and vulnerable risk groups |
| Israel | https://onlinelibrary.wiley.com/doi/abs/10.1111/phn.12367 caries risk assessment tool for public health nurses |
| Puerto Rico | Puerto Rico has a health reform program that provides medical and dental coverage for the Medicaid and Medicare-eligible and the medically indigent (federal poverty level below 200 %) populations, Law: 'Ley Núm. 11 de 23 de Junio de 1976, 'Ley de Reform Integral de Los Services de Salud de Puerto Rico'. Government Health Insurance: regular dental check-up, fluoride varnish application (every 6 months), restorations, dental surgery, sealants. |
| Sweden | In Sweden, all children are entitled to regular and complete dental care according to the Dental Care Act in Sweden the 21 county councils are responsible for carrying out dental care for children up to 24 years of age. How the county councils organize dental care for children differs and cannot be answered on a national level. The following questions are therefore not relevant to be answered on a national level but can be answered by each county council: - Does your country have specific programs that target the oral health care needs of socially disadvantaged child populations? - Which of the following is/are addressed by your country oral health policy/ies? Check all that apply the same applies for the questions on the National Universal Healthcare plan. Those cannot be answered on a national level. Dental care to children is regulated through the Dental Care Act but the county councils are responsible for implementation and delivering of dental care to the citizens. Specific programs that target different groups is thus organized and delivered by the county councils. |
| Montserrat | Oral health care is free to designated groups of people in Montserrat. This includes children right up to tertiary level education. We do not necessarily have a universal health system. The Government dental service is entirely free but only to specific groups of persons in the community. |
| Mexico | Mexico has changed in the last 2 years. Government has reduce the economic support for health (general health),eliminate the Seguro popular (an important social health program) and has intended to change for other one but it hasn´t worked well. The oral health program has disappeared in the fact. |
|  | Some plans exist in paper but not in daily reality. |
| Slovakia | In the present time, new government starts to prepare National healthcare program, which also include oral health care |
| Thailand | There are specific programs for special need child population provided in visual impairments group and children with cleft lip palate. |
| Serbia | Government surveillance system on oral health was active with the efforts of School of Dentistry University of Belgrade staff, in periods 1996-2000, and from 2009-2015 (second time without evaluation), The design of new program and preventive strategy is currently on going. |
| Jordan | All children less than 6 years in Jordan are covered both medically and dentally, including refugees. |
| Canada | In Canada health care delivery is the responsibility of Canada's Provincial and Territorial governments. Therefore, provincial and territorial oral health policies are captured in this survey.  The federal government is responsible for oral health care for those in the Canadian military, Registered First Nations and Inuit Peoples, and those in federal prisons. |
| Bangladesh | Oral Health Police is still neglected in Bangladesh, Need Improvement. |
| Switzerland | Each Kanton regulates itself of the health law whether there is school dental care or not. This then begins, if it exists, at the age of 4. There are individual Kanton’s that start dental health prevention earlier. Switzerland’s system still consists of basic health insurance and disability insurance. However, caries prevention is excluded from this. |
| South Africa | Although oral health policy exists in South Africa, it is very old and required an urgent update. There is also a degree of lack of implementation of these oral health policies and national frameworks for oral health. More dedicated leadership is required to manage oral health at district and provincial public health level. Moreover, dedicate implementation concurrent with monitoring and evaluation of oral health services and programs is required. |
| USA | The national health insurance plan only is available for low-income families and disadvantaged vulnerable communities is not for the overall general population |
| Spain | Epidemiological surveillance in oral health is not carried out by the health authorities. For the past 30 years it has been run by the General Dental Council. At present we do not have a Chief Dental Officer of the Spanish government. These functions are being performed by an official of the Ministry of Health who is a pharmaceutical professional. |
| France | Accessibility to dental care (curative mainly) is financially ensured as the national public insurance fund covers the whole population for dental care. Dental care is mainly provided by private practitioners (90%) who have to apply fixed or regulated fees to their patients. There are no dental hygienists nor dental nurses. Oral health promotion (OHP) is not organized at a national level with a dedicated plan. OHPs initiatives depend on the regional or local level or on non-governmental bodies. The last epidemiological national survey on dental caries in children has been conducted in 2006. |
| Mongolia | Oral health care is provided by the private sector. District hospitals provide dental care from the state. |
| Hong Kong Special Administrative Region, China | For oral care to children, the School Dental Care Service covers all primary school children, but children with intellectual disabilities are also eligible for oral care up to 18 years old. For preschool children with intellectual disabilities, there is Special Oral Care Service with outreach examination and preventive fluoride application plus treatment in the Children's Hospital. There is also a time-limited 'Jockey Club Children Oral Health Project” providing outreach dental care to all kindergartens funded by the Government. |
| Philippines | Regarding the UHC, we still need to actively advocate and campaign for primary care, preventive and control measures for funding. Our division will also conduct strategic planning by middle of next year. |
| Sudan | There is a developed oral health policy by different experts in the fields of dental public health specialist but unfortunately it is not endorsed yet. |
