# Supplementary material for: A global survey of national oral health policies and its coverage for young children
Source: Front Oral Health. 2024 Apr 5;5:1362647. doi: 10.3389/froh.2024.1362647 (PMC11026553; doi:10.3389/froh.2024.1362647)
Supplement: Supplementary file 3 [file Datasheet3.pdf]

Oral Health Policy Survey

Dear Respected Doctors, although oral health continues to be a burden on both developing and developed countries, nevertheless, it gains the least attention when it comes to policy establishment and implementation. The following short survey was created by a group of volunteering dental professionals from around the globe with the aim of assessing the existing oral health policies to identify best practices and gaps on both national and international levels.

If you have any questions/comments please feel free to contact Balgis Gafffar (bgosman@iau.edu.sa).

Country	
Is your country a World Dental Federation (FDI) member?	☐ Yes<br>☐ No
Is your country a member of International Association of Pediatric Dentistry (IAPD)?	☐ Yes<br>☐ No
Does your country have an Oral Health Directorate/Division/chief dental officer within Ministry of Health (MoH)?	☐ Yes<br>☐ No
Does the country have a national oral health policy document OR position statement on oral health?	☐ Yes<br>☐ No
If no, does the country have an oral health policy as part of a general health policy document (national tax on sugars, obligatory dental check-up within pediatric visits etc...)?	☐ Yes<br>☐ No

Which of the following/s is/are addressed by the oral health policy/prevention program?	☐ Reduction of sugar consumption, including sugar tax<br><br>☐ Implementation of oral hygiene measures (such as brushing days within schools, Distribution of toothpaste and toothbrushes, public campaigns).<br><br>☐ Provision of fluoride varnish for children at risk for caries.<br><br>☐ Promotion of the first dental visit by 12 months of age.<br><br>☐ Interprofessional collaboration with non-dental primary care providers to include oral health screenings as part of overall child health assessments.<br><br>☐ Coordination with medical providers to facilitate dental screenings, counseling, and preventive procedures to infants.<br><br>☐ Prenatal mother oral health education.<br><br>☐ Access to oral health care for at-risk populations of children as apart of early childhood oral health promotion.<br><br>☐ Incorporating Individual Risk Assessment, Anticipatory Guidance and Self-Management Goals as part of diagnosis and treatment planning in a "Disease Prevention Management Model" framework.<br><br>☐ Implementing government surveillance systems focused specifically on dental caries in the primary dentition of infants and preschoolers (0-5 years) in all the country.<br><br>☐ Others (please specify)<br>---<br>---<br>---<br>---<br>---

	-- --
Taskforce (Personnel involved in Oral Health Promotion Programs)?	☐ Dental<br>☐ Health professionals
Is the dental care part of the country "universal health care system", is there a Universal Health Coverage (UHC) for pediatric dentistry?	☐ Yes<br>☐ No
Any comments or notes?	

Thank you
